# Microperimetry-Based Fixation Training in Patients with Age-Related Macular Degeneration (AMD)

**DOI:** 10.3390/jcm15072651

**Published:** 2026-03-31

**Authors:** Karolina Ciszewska, Mateusz Winiarczyk, Dagmara Winiarczyk, Jerzy Mackiewicz

**Affiliations:** 1 Department of Vitreoretinal Surgery, Medical University of Lublin, 20-059 Lublin, Poland; karro8@o2.pl (K.C.); jerzymackiewicz@umlub.pl (J.M.); 2 Department of Internal Diseases of Small Animals, University of Life Sciences of Lublin, 20-400 Lublin, Poland; winiarczykdm@gmail.com

**Keywords:** age-related macular degeneration, geographic atrophy, microperimetry, biofeedback training, fixation stability, bivariate contour ellipse area, vision-related quality of life, NEI-VFQ-25

## Abstract

**Background**: Age-related macular degeneration (AMD) is the primary cause of severe visual acuity loss in individuals over 60 with increasing prevalence. Currently, no effective treatments exist for geographic atrophy and macular scarring, highlighting the need for visual rehabilitation in these patients. Microperimetry offers functional assessment at any AMD stage and employs fixation training to help patients utilize the most effective retinal areas for vision. **Methods**: A prospective study involving 25 patients (50 eyes) aged 67 to 90. The MAIA II microperimeter assessed scotoma size and location, retinal sensitivity, macular integrity, fixation parameters (P1, P2, 63%BCEA, 95%BCEA), fixation stability, and preferred retinal locus. Quality of life was evaluated using the National Eye Institute Visual Function Questionnaire (NEI-VFQ-25). A subgroup with inactive AMD-related macular changes, either bilateral geographic atrophy (13 patients, 26 eyes) or bilateral scarring (12 patients, 24 eyes), was identified, all exhibiting bilateral absolute central scotomas of at least 2 degrees. Each patient completed 10 fixation training sessions with a microperimeter, training the eye with better acuity weekly. One-week post-training, a functional assessment was performed on both trained and untrained eyes. **Results**: Fixation training significantly improved best corrected visual acuity (BCVA) in trained eyes (mean change −0.14 logMAR, *p* < 0.001, large effect size) and also in fellow untrained eyes (−0.16 logMAR, *p* < 0.001). BNVA improved from 2.25 to 1.86 in trained eyes (*p* < 0.001) and from 2.96 to 2.76 in untrained eyes (*p* = 0.004). Fixation stability parameters improved significantly, including increases in P1 and P2 and reductions in Bivariate Contour Ellipse Area (BCEA). Quality of life measured using the NEI-VFQ-25 questionnaire improved significantly in 9 of 11 domains. **Conclusions**: Microperimetry may be a valuable tool for assessing visual function in AMD patients. Fixation training with the MAIA II microperimeter is both safe and effective for vision rehabilitation in those with geographic atrophy and macular scarring.

## 1. Introduction

Age-related macular degeneration (AMD) is a progressive, multifactorial neurodegenerative disease affecting the central retina. It accounts for approximately 8.7% of blindness cases worldwide and represents the third leading cause of irreversible vision loss in the adult population. Due to population aging, the global number of individuals affected by AMD is expected to increase from 196 million in 2020 to nearly 288 million by 2040 [1,2].

In advanced stages, AMD may lead to legal blindness and substantial socioeconomic burden. In Poland, approximately 33% of individuals registered as visually disabled have certified visual impairment. The economic impact of AMD is also considerable. In the United States alone, the direct annual cost of treating AMD is estimated at approximately USD 2.8 billion, and the total societal costs associated with the disease are expected to increase substantially in the coming decades [3,4].

AMD is traditionally classified into two main clinical forms: the atrophic (“dry”) form, which accounts for approximately 90% of cases, and the neovascular (“wet”) form, which affects about 10% of patients. More recently, AMD has also been categorized according to disease stages into early, intermediate, and late forms [5].

Loss of central retinal function leads to significant visual impairment and affects many everyday visual tasks. One of the earliest symptoms is deterioration of near vision, particularly affecting reading ability. Damage to the macula also disrupts fixation stability. In healthy individuals, fixation involves aligning the macula of both eyes on the observed object. In patients with macular pathology, however, central fixation becomes impossible, forcing the visual system to search for an alternative retinal locus for fixation through rapid and unstable eye movements. In addition, binocular visual function may be compromised, which further affects depth perception and spatial orientation. As a result, patients frequently experience reduced reading speed and difficulty understanding written text [6].

Therapeutic options for advanced AMD remain limited. While the neovascular form can be treated with intravitreal anti-VEGF injections, there is currently no widely effective treatment for the atrophic form. Both forms may eventually lead to macular scarring or advanced geographic atrophy, which result in irreversible central vision loss and the development of extrafoveal fixation strategies [6,7].

Because many patients develop permanent central vision loss, visual rehabilitation plays an important role in the management of advanced AMD. The primary goal of rehabilitation is not only to improve visual function but also to enhance patients’ quality of life and independence. Previous studies have demonstrated that visual rehabilitation can significantly improve several domains of visual functioning, including general vision, distance activities, near activities, and peripheral vision [8,9].

Microperimetry has emerged as a valuable tool both for functional retinal assessment and for visual rehabilitation. By mapping retinal sensitivity and fixation behavior, microperimetry allows clinicians to identify functional retinal areas outside the central scotoma that may serve as a new preferred retinal locus (PRL). Using biofeedback-based fixation training, patients can be guided to utilize these areas more efficiently, potentially improving visual performance and functional outcomes [10,11]. Visual rehabilitation strategies have therefore gained increasing importance in patients with advanced AMD, particularly in those with geographic atrophy or macular scarring where therapeutic options remain limited. Microperimetry provides a unique opportunity to assess functional retinal sensitivity and fixation behavior while simultaneously enabling targeted rehabilitation through biofeedback fixation training. By guiding patients to use a more optimal retinal locus outside the central scotoma, fixation training may improve visual performance and functional outcomes. However, the clinical effectiveness of this approach in advanced AMD remains insufficiently studied.

The aim of this study was to evaluate the effect of microperimetry-based fixation training on visual function and quality of life in patients with advanced AMD, including geographic atrophy and macular scarring.

## 2. Material and Methods

A total of 27 patients with bilateral AMD, treated at the Clinic of Retina and Vitreous Surgery between April 2017 and November 2021, were included in this prospective study. All patients signed an informed consent form to participate in the study. The study was approved by the Bioethics Committee of the Medical University of Lublin (Bioethics Committee Resolution No. KE-0254/80/2017). The study was conducted in accordance with the Declaration of Helsinki.

A full cycle of examinations was completed by 25 patients (50 eyes) over a period of 11 weeks. Two patients, due to general health problems, had to discontinue their participation in the fixation training. Their data were not included in the final analysis of the results. Inclusion and exclusion criteria are presented in Table 1.

### 2.1. Study Groups

The study included 25 patients (50 eyes) aged between 67 and 90 years (mean age 79.48 years), of which 52% (*n* = 13) were men aged between 67 and 90 years (M = 78.9 years, SD = 6.5 years), and 48% (*n* = 12) were women aged between 71 and 85 (M = 80.1 years, SD = 4.8 years). The mean age of the men did not differ significantly from that of the women, t_Welch_(21.93) = 0.51, *p* = 0.620, ${\hat{g}}_{Hedges}$ = 0.20.

Patients were divided into two groups depending on the macular pathology associated with AMD:

The group of patients with bilateral geographic atrophy included 13 individuals (26 eyes) and constituted 52% of the subjects. There were no significant differences in age between men (*n* = 7, M = 78.4 years, SD = 6.8 years) and women (*n* = 6, M = 80.0 years, SD = 5.2 years) with atrophy, t_Welch_(10.89) = 0.47, *p* = 0.650, ${\hat{g}}_{Hedges}$ = 0.24. Geographic atrophy in both eyes without features of the active form of AMD was demonstrated in all cases.

The group of patients with bilateral macular scarring consisted of 12 individuals (24 eyes) and accounted for 48% of the subjects. There were no significant differences in age between men (*n* = 6, M = 79.6 years, SD = 6.77 years) and women (*n* = 6, M = 80.2 years, SD = 4.8 years) with scarring, t_Welch_(9.04) = 0.20, *p* = 0.850, ${\hat{g}}_{Hedges}$ = 0.10. An inactive form of macular scarring was demonstrated in both eyes of all patients.

No significant differences were found in the time from disease diagnosis between patients with atrophy (*n* = 13, M = 11.4 years, SD = 5.3 years) and patients with scarring (*n* = 12, M = 13.9 years, SD = 8.3 years).

In all patients participating in the study, fixation training was conducted on the eye with better BCVA, and in cases of equal acuity, on the eye with better functional characteristics based on microperimetry examination. All 25 patients underwent the same cycle of 10 fixation trainings. One eye was trained in each patient. In 14 cases, the left eye was trained, and in 11 cases, the right eye. Both the 25 trained eyes—designated as the “trained group,” and the 25 accompanying (untrained) eyes—referred to as the “untrained group,” were subjected to full functional evaluation (Table 2).

### 2.2. Clinical Examination

The refraction of both eyes was examined using the RT-7000 (Tomey Corporation, Nagoya, Japan) autorefractor. The best corrected visual acuity for distance (BCVA) was determined from 4 m distance using standardized ETDRS (Early Treatment Diabetic Retinopathy Study) logMAR charts. The illumination of the charts was 160 cd/m^2^. The best corrected visual acuity for near (BNVA) was determined for each eye separately from a distance of 35 cm using reduced Snellen near charts. An ophthalmological examination was conducted using a slit lamp for both eyes with an assessment of the anterior segment of the eye and evaluation of the fundus. Spectral-domain optical coherence tomography (OCT) of the macula of both eyes was performed using the SOCT Copernicus HR (Optopol, Zawiercie, Poland) device. In certain cases, fluorescein angiography (FA), and fundus autofluorescence (FAF) (Spectralis, Heidelberg Engineering, Heidelberg, Germany) and/or angio-OCT (AngioVue, Optovue Inc., Freemont, CA, USA) were performed. Fundus photography of both eyes was obtained using the VISUCAM 500 device (Carl Zeiss Meditec, Dublin, CA, USA). A full microperimetric examination of both eyes was conducted using the MAIA II microperimeter (Macular Integrity Assessment—Centervue/iCare, Padua, Italy). All procedures were conducted by CK. Assessed parameters are summarized in Table 3.

The examinations were performed before the patient started the vision training, and also a week after completing the full cycle of training. The patient’s quality of life was assessed using the standardized “National Eye Institute Visual Functioning Questionnaire-25” (NEI-VFQ-25).

### 2.3. Microperimetry Training

Peripheral vision training was conducted using the training module of the MAIA microperimeter. Fixation training was carried out on the eye with better visual acuity (BCVA) for all patients participating in the study, and in cases of equal acuity. At baseline, the eye with better BCVA (or the eye with better functional characteristics based on the microperimetry examination in cases of equal acuity) was selected for fixation training, while the fellow eye served as an internal control. Therefore, baseline visual acuity was expectedly higher in the trained eyes compared with the fellow eyes. This design was used to maximize the rehabilitation potential while allowing within-patient comparison.

A new Preferred Retinal Locus (PRL), also known as the Target Preferred Retinal Locus (PRT) or the Fixation Training Target (FTT), was determined for each patient. Each fixation training session lasted 10 min. The target PRL was selected individually for each patient based on baseline microperimetry results. The selection was performed manually by the investigator using the MAIA II software 2.3 and retinal sensitivity maps. The training target was chosen outside the absolute central scotoma in a retinal area with relatively preserved sensitivity. When several potential areas were present, preference was given to locations as close as possible to the foveal center and oriented horizontally relative to the initial PRL, which facilitates everyday visual tasks such as reading. Upper retinal quadrants were generally preferred. Structural retinal integrity assessed using OCT and/or fundus autofluorescence was also considered when selecting the training target. Additional factors included the size and location of the central scotoma, baseline fixation characteristics, and BCEA parameters. During training, patients received real-time auditory and visual biofeedback signals from the device, allowing them to gradually shift fixation from the original PRL toward the selected PRT/FTT location.

Every patient underwent a cycle of 10 training sessions, conducted at weekly intervals. A week after the completion of the fixation training series, all examinations were repeated for each patient, and the quality of life was reassessed using the NEI-VFQ-25 questionnaire. During the microperimetry examination, the “follow-up” function was used to compare results with the pre-rehabilitation state. Subsequently, the results were subjected to statistical analysis.

### 2.4. Statistical Analysis

Analyses were conducted using the statistical software R (version 4.1.1; R Core Team, 2021). The significance level for statistical tests was set at α = 0.05. To calculate the differences between the mean measures of groups, the central measure of tendency was used. Parametric tests were employed for variables with normal distributions, while non-parametric tests were used for variables with distributions deviating from normal.

For variables with a normal distribution, mean measures (M) and standard deviations (SD) were reported. For distributions differing from normal, median (Mdn) and interquartile ranges (Q1–Q3) were reported. For hypothesis testing, the paired *t*-test was used for two groups and one variable with a normal distribution The magnitude of effect sizes based on Hedges’ g was interpreted according to Cohen’s conventional thresholds: small (0.2), medium (0.5), and large (0.8). Effect sizes based on Hedges’ g were reported as absolute values, where larger positive values indicate a stronger improvement effect.

For two groups and one variable with a non-normal distribution, the Wilcoxon signed-rank test was used, with the effect size calculated as a biserial rank correlation, interpreted according to Funder’s convention.

The dependency of variables was examined using Fisher’s exact test, with Cramer’s V calculated as a measure of association strength. For multivariate analysis with repeated measures, linear and mixed regression models were used to estimate the influence of independent variables on dependent variables, considering repeated measurements. 95% confidence intervals (CI) and *p*-values were calculated using the approximation of Wald’s t-distribution.

## 3. Results

### 3.1. Differences in Mean BCVA

A significant improvement in BCVA was demonstrated for all groups and conditions after 10 completed training sessions. Eyes with atrophy showed a more pronounced effect compared to eyes with scars. Improvements were also observed in fellow untrained eyes, particularly in patients with geographic atrophy.

The results of the assessment of intragroup differences in mean BCVA for six subgroups are presented in Table 4.

### 3.2. Differences in Mean BNVA

A statistically significant improvement in BNVA after 10 sessions was observed in all groups except for the untrained eye group with macular scars (t = 1.63, *p* = 0.130, effect size = 0.44). Patients with atrophy showed a more pronounced effect compared to patients with scars. The untrained eye (untrained group) of the subjects also improved, although to a lesser extent compared to the trained eye (smaller effect size). The results of the assessment of intragroup differences in mean BNVA for six subgroups are presented in Table 5.

### 3.3. Differences in Mean P1 and P2

**Figure 1:**
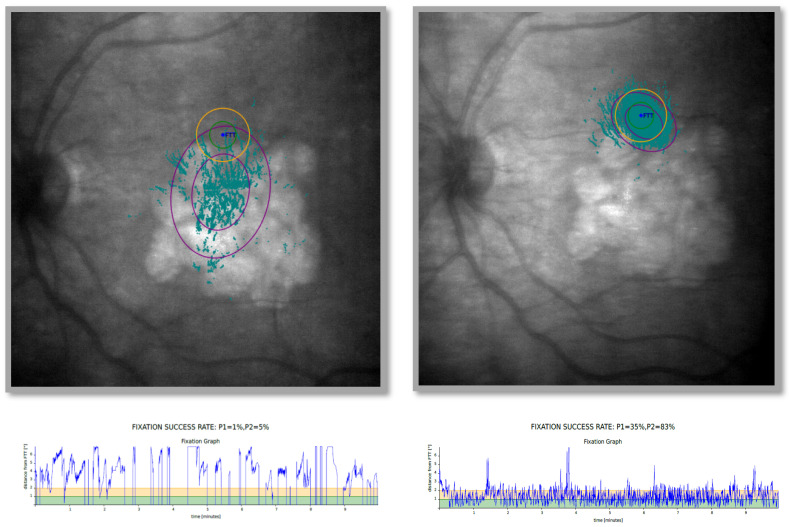
The results of microperimetry tests showing the effects of fixation training in terms of P1 and P2 in a selected patient.

There was a significant increase in P1 with a very large effect size after 10 completed training sessions. The significance of the effect was noted for all groups except for the trained group with scars. A significant increase in P2 was demonstrated for all groups and conditions after 10 completed training sessions. The magnitude of the effect obtained for both atrophy and scars was similar. In several parameters, improvements were also observed in the fellow untrained eyes, suggesting a task-learning effect, or a possible binocular transfer effect. P1 and P2 intragroup changes are presented in Table 6 and Table 7. For normally distributed variables, paired *t*-test and Hedges’ g were used. For non-normally distributed variables, Wilcoxon signed-rank test and rank-biserial correlation were used. In Figure 1 pre and post training P1 and P2 location is presented.

### 3.4. Differences in BCEA

Fixation training produced a substantial reduction in both 63% BCEA and 95% BCEA values, reflecting a marked improvement in fixation precision. In trained eyes, 63% BCEA decreased from 18.9 deg^2^ to 10.0 deg^2^ and 95% BCEA from 56.7 deg^2^ to 29.9 deg^2^. This represents a nearly 50% reduction in the area encompassing the majority of fixation points, indicating that post-training fixation was more tightly clustered around the preferred retinal locus (PRL). In fellow eyes, despite the absence of direct training, 63% BCEA decreased from 24.2 deg^2^ to 17.4 deg^2^ and 95% BCEA from 73.6 deg^2^ to 59.6 deg^2^, suggesting partial transfer of fixation stability improvements through binocular visual processing and cortical adaptation. Subgroup analysis revealed greater BCEA reduction in geographic atrophy (GA) compared to macular scarring, consistent with the hypothesis that retinal integrity influences the capacity to refine fixation.

BCEA detailed data can be found in the Supplementary Materials.

### 3.5. NEI-VFQ-25 Questionnaire Results

10 training sessions allowed for a significant improvement in 9 out of 11 components of the NEI-VFQ-25 questionnaire with a large effect size. No statistically significant improvement was obtained in the areas of General Health and Eye Pain. A greater degree of improvement was characterized by patients with scars.

The total NEI-VFQ-25 score for all patients increased from a value of 40.91 before the training to 54.96 after the training, including for patients with atrophy from 43.54 to 57.2, and for patients with scars from 37.87 to 52.55. Full NEI-VFQ-25 results can be found in the Supplementary Materials.

## 4. Discussion

The findings from this study indicate that fixation training using microperimetry significantly enhances visual acuity in patients with advanced AMD, including those with geographic atrophy and macular scarring. Notably, the improvement in best-corrected visual acuity (BCVA) was observed across all patients, demonstrating a large effect size. Specifically, post-training BCVA showed a marked improvement of −0.14 logMAR. This enhancement was more pronounced in eyes with geographic atrophy compared to those with macular scarring. The results support previous findings by Estudillo et al. and Oflaz et al., which also reported significant BCVA improvements following similar training regimens. These consistent outcomes underscore the potential of microperimetry-based fixation training as a viable rehabilitation method to mitigate the visual deficits associated with AMD.

### 4.1. Impact on BCVA

Microperimetry-based fixation training was associated with a significant improvement in BCVA in both trained and fellow untrained eyes. These findings suggest that fixation training may enhance visual performance not only through local retinal adaptation but also through mechanisms related to binocular processing and cortical plasticity. Estudillo et al., in a prospective study involving 18 patients with geographic atrophy due to AMD, used a similar regimen of 10-min fixation training sessions with the MAIA microperimeter. They found an improvement in BCVA from 0.7 to 0.6 logMAR [12]. Similarly, a study by Oflaz et al. in the Turkish Journal of Ophthalmology reported significant BCVA improvement in 29 AMD patients after 8 fixation training sessions, although they did not differentiate between atrophy and scarring effects [13].

The literature indicates the utility of microperimetry-based vision rehabilitation in various ophthalmic conditions, including nystagmus [14], amblyopia, glaucoma [15,16], albinism [17], Stargardt disease [18,19], myopic maculopathy [18,20,21], central serous retinopathy [22], traumatic scars [13,18,23], and post-surgical states of macular holes [24,25] and retinal detachments [26]. However, fewer studies focus on AMD rehabilitation using the MAIA microperimeter. Notable examples include the works by Estudillo et al. [12] and Oflaz et al. [13], as well as a 2021 study by Sahli et al. reporting positive outcomes in 17 AMD patients using MAIA [19].

Other microperimeters like MP-1 and MP-3 also feature vision rehabilitation software. Studies have documented the impact of fixation training using the MP-1 in AMD patients, primarily analyzing BCVA. For instance, Vingolo et al. reported BCVA improvement in all 15 AMD patients after 10–12 MP-1 training sessions and suggested that reminder sessions every 3 months could help maintain visual function [27,28]. A comprehensive study by Pacella et al. involving 122 AMD patients undergoing 16 MP-1 training sessions found significant BCVA improvement in 92 eyes, with follow-up assessments showing some decline in BCVA after 6 and 12 months, primarily due to AMD progression [29].

Tarita-Nistor et al. [30], Amore et al. [31], and Ratra et al. [18] also reported BCVA improvements in AMD patients following microperimetry-based rehabilitation. A 2022 study by Chinese researchers documented positive rehabilitation outcomes using the MP-3 microperimeter, showing BCVA improvement in all participating AMD patients [32].

### 4.2. Impact on BNVA

Visual rehabilitation with MP in our study was associated with a clinically and statistically significant improvement in best near visual acuity (BNVA) in most analyzed groups. Improvements in near vision are particularly relevant for daily functioning, as reading ability represents one of the most important visual tasks affected in advanced AMD. Clinically, it is notable that in two patients who were unable to read at baseline, BNVA improved to 3.0, effectively restoring the ability to read large print and perform near tasks. In reading, approximately 95% of the time is devoted to fixation pauses, so improved fixation stability has a direct functional impact on near vision.

The modest yet noticeable improvement in fellow eyes is attributed either to the task-learning effect, or to the binocular nature of vision and potential neuroplasticity, allowing partial transfer of acquired fixation strategies to the non-trained eye. These observations are consistent with the previous literature: Tarita-Nistor et al. reported a two-line BNVA gain following biofeedback [30], while Vingolo et al. demonstrated a significant reduction in the minimum readable print size [28]. In studies assessing reading speed instead of BNVA (e.g., MNREAD), improvements were observed from 47 to 69 words/min in GA patients [12] and from 38 to 45 words/min [13]. However, the lack of a standardized Polish reading test limits direct comparison of BNVA with reading performance. In summary, experience gained through microperimetry-based fixation training leads to tangible BNVA improvements most pronounced in trained eyes and in patients with GA, with the effect size reflected in both statistical measures (*t*-tests, mixed models) and practical functional outcomes such as regaining the ability to read large prints.

### 4.3. Effect on Fixation Stability (P1, P2 and BCEA)

Fixation stability parameters (P1 and P2) also improved following the training sessions, indicating better control of extrafoveal fixation. Improved fixation stability may allow patients to use the preferred retinal locus more efficiently, which in turn may contribute to better visual performance. Similar findings have been reported in previous studies evaluating biofeedback-based rehabilitation in patients with central vision loss.

Comparable observations have been reported in other studies, which attributed improvements in fellow eyes to binocular integration and cortical reorganization [13,18,30]. However, an alternative explanation for the improvement observed in untrained eyes may be a task-learning effect related to repeated exposure to the microperimetry examination. With repeated testing, patients may become more familiar with the procedure and the auditory feedback signals used during fixation assessment, which could partially improve test performance independently of true functional retinal changes. Therefore, although binocular transfer and cortical adaptation remain plausible mechanisms, the contribution of procedural learning should also be considered when interpreting the improvements observed in untrained eyes.

The magnitude of BCEA reduction in our cohort, approaching 50% in trained eyes, lies at the upper range of previously published results, suggesting that the training protocol was particularly effective in promoting tighter clustering of fixation points. More substantial changes in geographic atrophy compared to scarring may further support the role of preserved retinal architecture in enabling PRL refinement and improved fixation stability, consistent with observations reported by other groups [12,32].

Functionally, higher P1/P2 values and reduced BCEA indicate improved fixation stability, which minimizes retinal image motion and optimizes spatial resolution. This is particularly important for near-vision tasks such as reading, where fixation pauses account for approximately 95% of total reading time; therefore, even modest improvements in fixation stability may translate into meaningful functional benefits.

### 4.4. Effect on the Quality of Life

Visual rehabilitation also translated into improvements in several domains of the NEI-VFQ-25 questionnaire, suggesting a positive impact on patients’ perceived visual functioning and daily activities. Enhancements in BCVA, BNVA, and fixation stability (P1, P2, BCEA) translate into more efficient and reliable visual processing, particularly for tasks such as reading, recognizing faces, and navigating unfamiliar environments. These functional improvements reduce the visual effort required for daily activities, thereby increasing independence and confidence.

The larger quality of life gains in patients with macular scarring compared to those with geographic atrophy may reflect a greater perceived benefit when baseline visual function is more compromised, even if absolute visual acuity changes are similar.

Improvements in quality of life are particularly important in advanced AMD, where therapeutic options remain limited and rehabilitation plays a key role in maintaining independence.

### 4.5. Limitations of the Study

It is important to acknowledge the limitations of the presented study. This study has several limitations that should be acknowledged. Firstly, it is a pilot study with a relatively small sample size, and the absence of a suitable control group limits the generalizability of the findings. The small number of participants may affect the statistical power of the study and the robustness of the conclusions drawn. Another limitation is the lack of long-term follow-up to assess the persistence of training effects. Some authors have conducted evaluations months after the training, but our plans were disrupted by the COVID-19 pandemic, although the question should be raised of how much a delayed analysis might be affected by the natural progression of the disease. Like Estudillo et al. and Oflaz et al., we assessed the effects of vision rehabilitation a week after the training cycle, without long-term evaluation. These authors emphasize that with an 8–10 training session scheme, the results can be attributed to the rehabilitation method itself, with minimal disease progression impact over such a short period [12,13]. However, long-term studies could be useful, and implementing reminder training sessions could help maintain therapy effects.

Another limitation is the relatively short follow-up period. Functional outcomes were assessed only one week after completion of the training cycle. In a progressive condition such as AMD, the long-term persistence of rehabilitation effects remains uncertain. Future studies with extended follow-up periods, for example at 3- or 6-month intervals, would be valuable to determine whether the improvements in visual acuity and fixation stability observed in this study are maintained over time.

Additionally, noting the size of the central scotoma in square degrees (deg^2^) would be valuable to identify patients most responsive to vision rehabilitation. The literature suggests that improvement in fixation post-training might be limited in patients with central scotomas larger than 20 deg^2^ [33]. However, there are studies documenting the effectiveness of microperimetry training for both very small [34] and large scotomas [27]. Morales et al. even note that positive fixation training effects are achievable regardless of scotoma size [22]. Further studies with larger patient groups and varying disease stages, along with long-term follow-up, are necessary to supplement a more precise functional assessment of fixation training effects.

## 5. Conclusions

Microperimetry is a valuable method for visual rehabilitation of patients with AMD. In patients with geographic atrophies and scars in the macula due to AMD, completing a series of fixation training using microperimetry results in improved visual acuity for distance and near vision for both eyes. Greater improvement in visual acuity for distance and near vision as a result of the described vision rehabilitation method occurs in patients with geographic atrophy compared to patients with scars in the macula.

As a result of the application of peripheral vision training, there is an improvement in selected fixation parameters of the trained eye and the fellow eye. The improvement in selected fixation parameters is similar in cases of atrophy and scars. Fixation training does not seem to have a significant impact on retinal sensitivity. The quality of life of patients with AMD significantly improves thanks to peripheral vision training.

Collectively, these results support the incorporation of fixation stability metrics into standard rehabilitation outcome assessments and reinforce the dual role of microperimetry as both a precise diagnostic tool and a targeted intervention platform in late-stage AMD.

## Figures and Tables

**Table 1:** Inclusion and exclusion criteria for the study.

Inclusion Criteria	Exclusion Criteria
Presence of bilateral absolute central scotoma on the background of AMD with a diameter of at least 2 degrees. Presence of inactive changes related to AMD in the macula—bilateral dominant geographic atrophy or bilateral scar. BCVA of the better eye not worse than 1.7 on the logMAR scale Age > 45 years Patient’s informed written consent to participate in an 11-week research project.	Signs of active disease that could qualify the patient for anti-VEGF drug injections. Receiving anti-VEGF drug injections within the last 6 months Significant opacities in the optical centers, such as keratopathies, cataracts, and vitreous opacities High refractive errors (above 5 diopters spherical or above 2.5 diopters cylindrical) Glaucoma and other active ophthalmic diseases (especially affecting the retina, choroid, and optic nerve) that could affect retinal sensitivity. Past surgeries on the eyeball (excluding injections and cataract surgery) Hearing disorders that would prevent the patient from receiving auditory signals sent by the microperimeter. Diabetes and other general diseases that could affect the course of the study; arterial hypertension was not included in the exclusion criteria if it did not result in hypertensive retinopathy. The patient’s general condition, including motor disorders, preventing the performance of examinations.

**Table 2:** Eyes included in the study.

	Macular Atrophy	Macular Scarring	Total
Untrained group (fellow eyes)	13	12	25
Trained group	13	12	25
Eyes, total	26	24	50

**Table 3:** Parameters assessed in microperimetry examination.

Parameters Assessed in Microperimetry Examination
retinal sensitivity map average retinal sensitivity (AT) size and location of scotoma macular integrity (MI) fixation parameters in the form of P1 and P2 as well as 63%BCEA and 95%BCEA fixation stability group (stable/relatively unstable/unstable) preferred retinal locus (PRL)

**Table 4:** Differences in mean BCVA.

Macula Status	Group	n_pairs_	Mean BCVA		t_-statistic_	${\hat{g}}_{Hedges}$	Effect Size	*p*
			Before Training	After 10 Sessions				
Atrophy + Scar	Trained	25	0.98 (0.34)	0.84 (0.32)	6.82	1.32	Large	**<0.001**
Atrophy + Scar	Untrained	25	1.43 (0.25)	1.27 (0.28)	6.35	1.23	Large	**<0.001**
Atrophy	Trained	13	0.87 (0.34)	0.72(0.29)	6.32	1.64	Large	**<0.001**
Atrophy	Untrained	13	1.35 (0.23)	1.17 (0.25)	4.74	1.23	Large	**<0.001**
Scar	Trained	12	1.11 (0.30)	0.97 (0.30)	3.74	1.00	Large	**0.003**
Scar	Untrained	12	1.51 (0.25)	1.38 (0.29)	4.49	1.20	Large	**<0.001**

**Table 5:** Differences in mean BNVA.

Macula Status	Group	n_pairs_	Mean BNVA		t_-statistic_	${\hat{g}}_{Hedges}$	Effect Size	*p*
			Before Training	After 10 Sessions				
Atrophy + Scar	Trained	25	2.25 (0.84)	1.86 (0.84)	4.93	0.95	Large	**<0.001**
Atrophy + Scar	Untrained	25	2.98 (0.59)	2.76 (0.73)	3.23	0.62	Medium	**0.004**
Atrophy	Trained	13	1.96 (0.89)	1.58 (0.89)	3.99	1.03	Large	**0.002**
Atrophy	Untrained	13	3.0 (0.56)	2.71 (0.78)	2.84	0.74	Medium	**0.010**
Scar	Trained	12	2.56 (0.68)	2.17 (0.70)	2.99	0.80	Large	**0.010**
Scar	Untrained	12	2.96 (0.65)	2.81 (0.70)	1.63	0.44	Small	0.130

**Table 6:** Differences in mean P1.

Macula Status	Group	n_pairs_	Mean P1		Wilcoxon V	${\hat{g}}_{Hedges}$	Effect Size	*p*
			Before Training	After 10 Sessions				
Atrophy + Scar	Trained	25	20.0 (9.0–28.0)	29.0 (20.0–35.0)	53.0	0.67	V. large	**0.003**
Atrophy + Scar	Untrained	25	14.0 (8.0–21.0)	20.0 (14.0–39.0)	45.0	0.72	V. large	**0.002**
Atrophy	Trained	13	15.0 (8.0–27.0)	26.0 (21.0–32.0)	16.0	0.65	V. large	**0.040**
Atrophy	Untrained	13	15.0 (10.0–19.0)	23.0 (19.0–36.0)	15.0	0.67	V. large	**0.040**
Scar	Trained	12	23.5 (15.8–28.8)	30.0 (19.8–40.8)	13.0	0.67	V. large	0.050
Scar	Untrained	12	13.5 (7.8–21.5)	18.5 (13.8–48.3)	8.5	0.78	V. large	**0.020**

**Table 7:** Differences in mean P2.

Macula Status	Group	n_pairs_	Mean P2		t_-statistic_	${\hat{g}}_{Hedges}$	Effect Size	*p*
			Before Training	After 10 Sessions				
Atrophy + Scar	Trained	25	50.2 (23.8)	67.1 (17.6)	−4.6	0.89	Large	**<0.001**
Atrophy + Scar	Untrained	25	43.8 (23.5)	55.8 (26.2)	−5.02	0.97	Large	**<0.001**
Atrophy	Trained	13	47.6 (25.1)	65.0 (18.0)	−3.32	0.86	Large	**0.006**
Atrophy	Untrained	13	45.0 (24.5)	58.2 (24.5)	−3.75	0.97	Large	**0.003**
Scar	Trained	12	53.0 (22.9)	69.3 (17.7)	−3.04	0.82	Large	**0.010**
Scar	Untrained	12	42.5 (23.2)	53.2 (28.8)	−3.22	0.87	Large	**0.008**

## Data Availability

The raw data supporting the conclusions of this article will be made available by the authors on request.

## Supplementary Materials

The following supporting information can be downloaded at: https://www.mdpi.com/article/10.3390/jcm15072651/s1.
