# Supplementary material for: Microperimetry-Based Fixation Training in Patients with Age-Related Macular Degeneration (AMD)
Source: J Clin Med. 2026 Mar 31;15(7):2651. doi: 10.3390/jcm15072651 (PMC13073798; doi:10.3390/jcm15072651)
Supplement: Supplementary file 1 [file jcm-15-02651-s001.zip › Suppl. Table S1 and Figure S1.pdf]

Supplementary Table S1 and Supplmentary Figure S1. Assessment of the significance of intragroup differences in mean 63%BCEA and 95%BCEA before and after training.

Choroba	*Grupa*	n_{pairs}	*Średnie miary 63%BCEA*		$V_{Wilcoxon}$	$\hat{r}^{rank}_{biserial}$	*Efekt*	*p*
			Przed treningami	*Po 10 treningach*				
Zanik + Blizna	Trenowana	25	18.9 (11.9-30.4)	10.0 (6.9-16.4)	284.5	0.75	B.duży	**0.001**
Zanik + Blizna	Nietrenowana	25	24.2 (15.8-38.7)	17.4 (7.3-28.4)	292.5	0.80	B.duży	**0.001**
Zanik	Trenowana	13	19.8 (12.4-31.9)	10.0 (7.0-15.7)	79.0	0.74	B.duży	**0.020**
Zanik	Nietrenowana	13	24.1 (15.8-32.2)	17.3 (10.8-26.9)	83.0	0.82	B.duży	**0.009**
Blizna	Trenowana	12	15.1 (11.0-30.3)	10.6 (6.3 -16.7)	69.0	0.77	B.duży	**0.020**
Blizna	Nietrenowana	12	32.0 (15.9-40.1)	21.8 (7.2-29.9)	68.5	0.76	B.duży	**0.020**

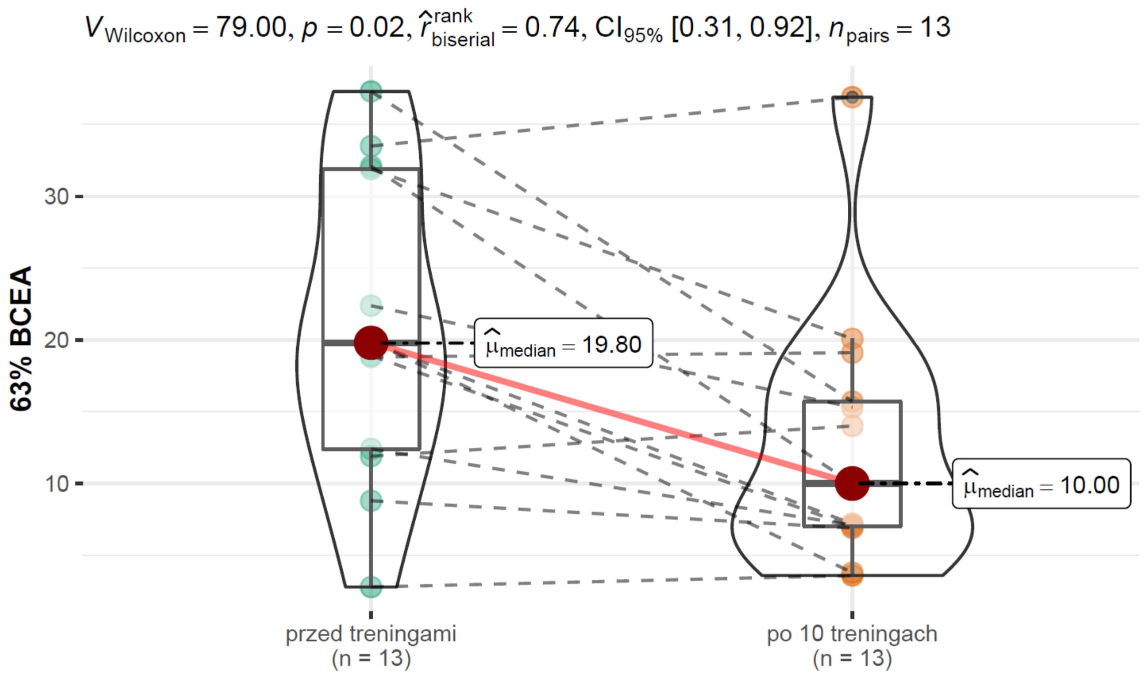

Results of the assessment of intragroup mean 63%BCEA for the Geographic Atrophy group (trained eyes).

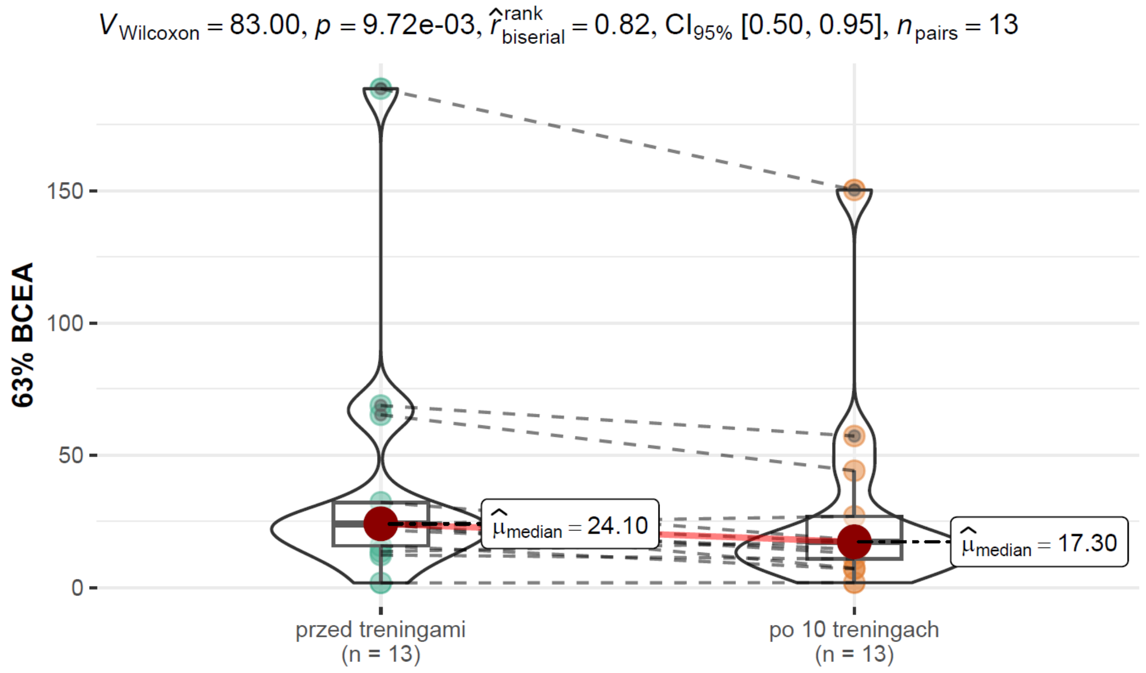

Results of the assessment of intragroup mean 63%BCEA in the Geographic Atrophy group (untrained eyes).

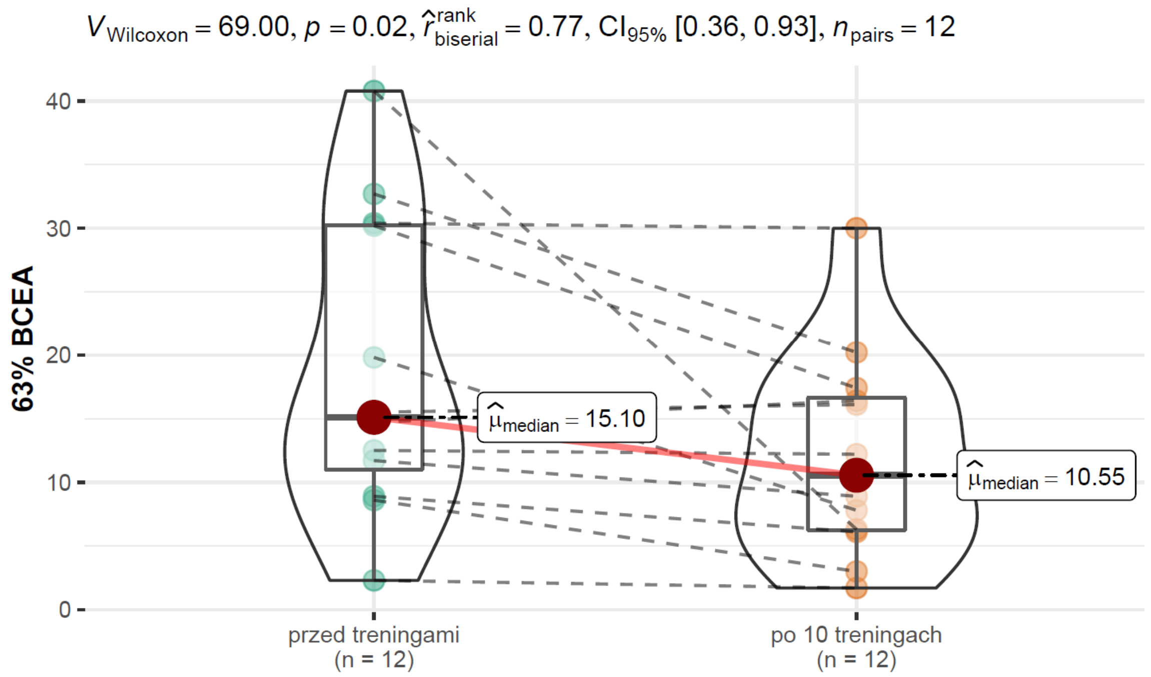

Results of the assessment of intragroup mean 63%BCEA in the Macular Scarring group (trained eyes).

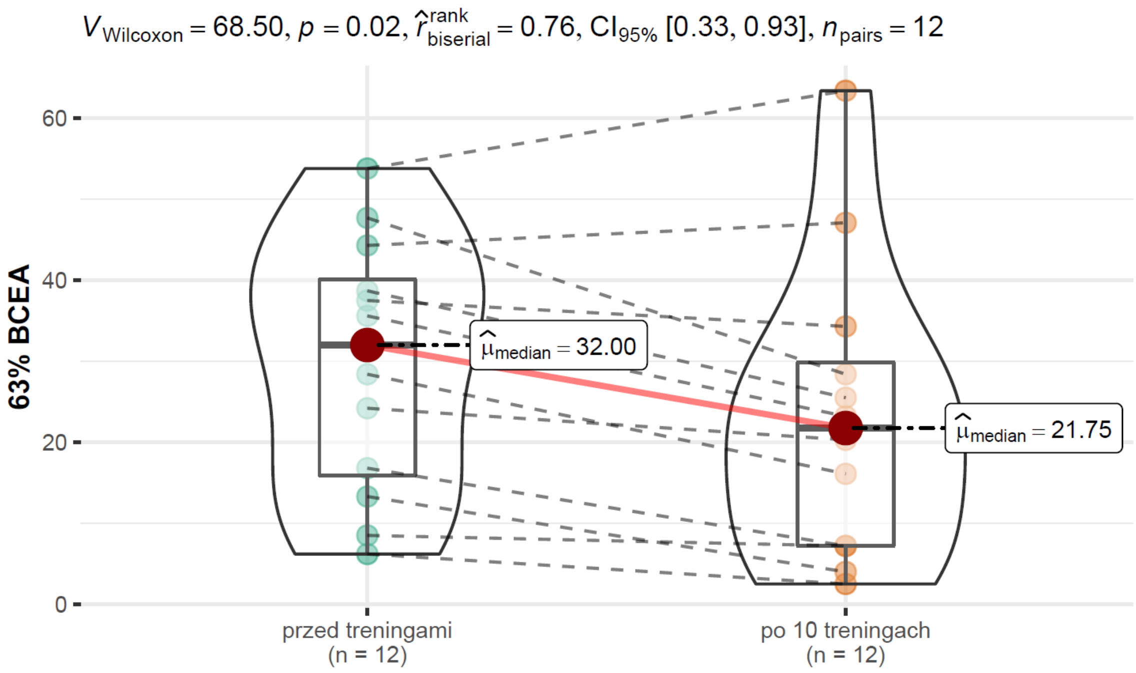

Results of the assessment of intragroup mean 63%BCEA in the Macular Scarring group (untrained eyes).

Statistical test results for the assessment of intragroup mean 95%BCEA differences, categorized by study group and disease type.

Choroba	*Grupa*	n_{pairs}	*Średnie miary 95%BCEA*		$V_{Wilcoxon}$	$\hat{r}^{rank}_{biserial}$	*Efekt*	*P*
			Przed treningami	*Po 10 treningach*				
Zanik + Blizna	Trenowana	25	56.7 (37.5-94.4)	29.9 (19.7-49.2)	285.0	0.75	B.duży	**0.001**
Zanik + Blizna	Nietrenowana	25	73.6 (47.2-112.3)	59.6 (32.5-98.3)	290.0	0.78	B.duży	**<0.001**
Zanik	Trenowana	13	56.7 (39.7-95.4)	29.9 (20.6-47.1)	79.0	0.74	B.duży	**0.020**
Zanik	Nietrenowana	13	72.2 (47.2 -95.5)	52.0 (32.5-95.5)	83.0	0.82	B.duży	**0.009**
Blizna	Trenowana	12	41.6 (36.9-92.0)	31.7 (19.5-50.3)	70.0	0.79	B.duży	**0.020**
Blizna	Nietrenowana	12	84.5 (61.5-114.8)	72.9 (43.0-99.1)	66.0	0.69	B.duży	**0.040**

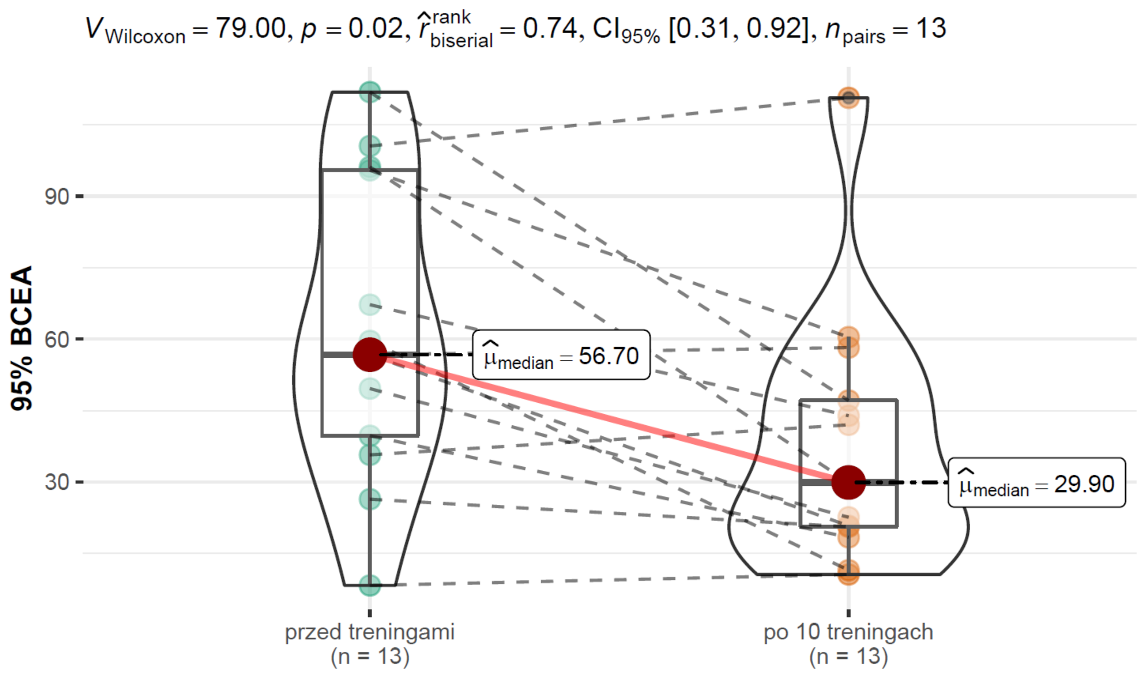

Results of the assessment of intragroup mean 95%BCEA in the Geographic Atrophy group (trained eyes).

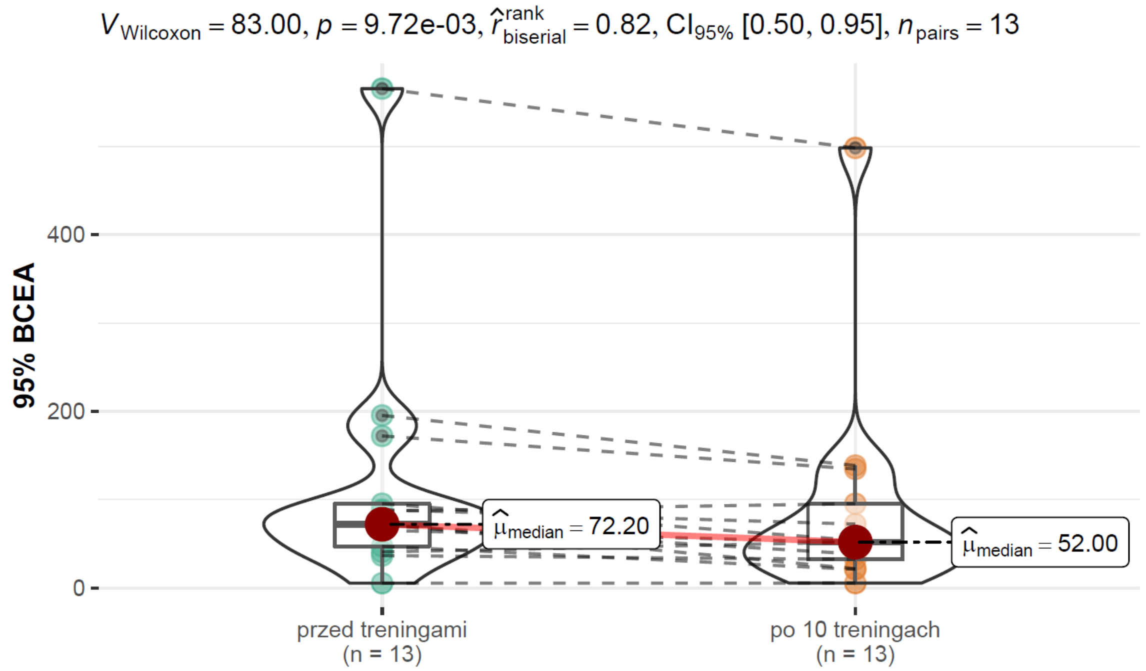

Results of the assessment of intragroup mean 95%BCEA in the Geographic Atrophy group (untrained eyes).

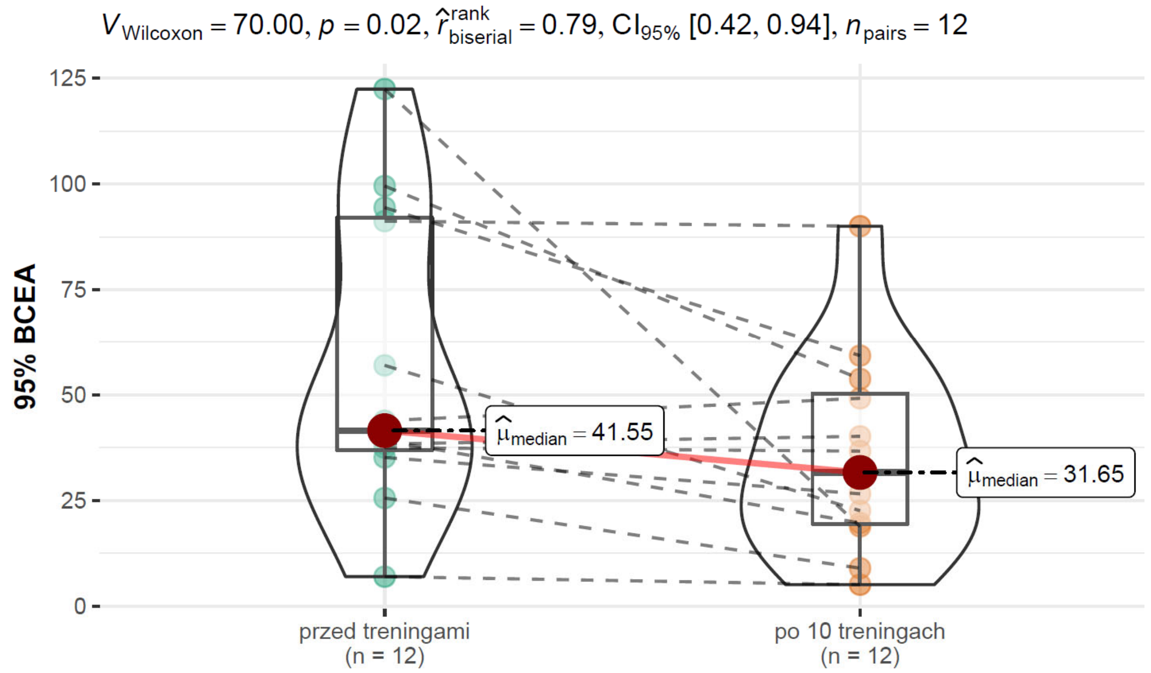

Results of the assessment of intragroup mean 95%BCEA in the Macular Scarring group (trained eyes).
