# Supplementary material for: Microperimetry-Based Fixation Training in Patients with Age-Related Macular Degeneration (AMD)
Source: J Clin Med. 2026 Mar 31;15(7):2651. doi: 10.3390/jcm15072651 (PMC13073798; doi:10.3390/jcm15072651)
Supplement: Supplementary file 1 [file jcm-15-02651-s001.zip › Supplementary table S2.pdf]

Supplementary Table S2. *Analysis of the NEI-VFQ-25 questionnaire results across different dimensions and groups.*

	Disease	n_{pairs}	*Mean values 95%*		*Test*	*Test statistics*	*ES*	*Effect*	*P*
			Before training	*After 10 sessions*					
General health	Atrophy+Scar	25	35.7 (10.2)	39.2 (11.7)	t Student	-1.78	-0.35	Small	0.090
	Atrophy	13	37.7 (11.4)	40.4 (13.8)		-0.82	-0.21	Small	0.430
	Scar	12	33.5 (8.7)	37.9 (9.3)		-2.01	-0.54	Medium	0.070
General vision	Atrophy+Scar	25	37.0 (8.3)	52.6 (12.1)	t Student	-8.51	-1.65	Large	**<0.001**
	Atrophy	13	39.6 (6.9)	53.5 (12.5)		-5.59	-1.45	Large	**0.001**
	Scar	12	34.2 (9.0)	51.7 (12.3)		-6.44	-1.73	Large	**<0.001**
Ocular pain	Atrophy+Scar	25	75.0 (21.0)	79.5 (18.7)	t Student	-1.40	-0.27	Small	0.170
	Atrophy	13	76.9 (19.7)	83.7 (15.6)		-1.10	-0.29	Small	0.290
	Scar	12	72.9 (23.1)	75.0 (21.3)		-1.48	-0.40	Small	0.170
Near activities	Atrophy+Scar	25	25.0 (16.7-25.0)	29.2 (20.8-37.5)	Wilco-xon	0	-1.0	Large	**<0.001**
	Atrophy	13	25.0 (20.8-33.3)	37.5 (25.0-45.8)		0	-1.0	Large	**0.002**
	Scar	12	18.8 (12.5-25.0)	27.1 (19.8-34.3)		0	-1.0	Large	**0.004**
Distance activities	Atrophy+Scar	25	33.7 (17.2)	45.3 (17.0)	t Student	-8.78	-1.70	Large	**<0.001**
	Atrophy	13	36.7 (17.1)	46.7 (18.4)		-6.34	-1.65	Large	**<0.001**

	Blizna	12	30.4 (17.5)	43.8 (16.0)		-6.31	-1.70	Large	**<0.001**
Social life	Atrophy+Scar	25	44.3 (18.3)	56.0 (18.6)	t Student	-6.72	-1.30	Large	**<0.001**
	Atrophy	13	46.2 (21.4)	57.1 (22.5)		-4.25	-1.10	Large	**0.001**
	Scar	12	42,3 (14.9)	54.9 (14.0)		-5.20	-1.39	Large	**<0.001**
Meantal health	Atrophy+Scar	25	26.0 (15.3)	47.4 (18.9)	t Student	-6.72	-1.30	Large	**<0.001**
	Atrophy	13	25.4 (14.9)	48.1 (20.4)		-4.95	-1.28	Large	**<0.001**
	Scar	12	26.7 (16.3)	46.7 (17.8)		-4.38	-1.18	Large	**<0.001**
Role difficulties	Atrophy+Scar	25	37.8 (23.2)	52.0 (16.8)	t Student	-5.72	-1.11	Large	**<0.001**
	Atrophy	13	40.9 (25.6)	54.8 (17.9)		-3.00	-0.78	Medium.	**0.010**
	Scar	12	30.2 (20.1)	49.0 (15.8)		-5.91	-1.59	Large	**<0.001**
Dependance	Atrophy+Scar	25	44.5 (16.7)	64.5 (16.2)	t Student	-8.26	-1.60	Large	**<0.001**
	Atrophy	13	47.6 (17.8)	66.4 (18.9)		-5.10	-1.32	Large	**<0.001**
	Scar	12	41.2 (15.4)	62.5 (13.3)		-6.64	-1.78	Large	**<0.001**
Color vision	Atrophy+Scar	25	48.0 (17.6)	62.0 (21.8)	t Student	-5.53	-1.07	Large	**<0.001**
	Atrophy	13	50.0 (20.4)	65.4 (21.7)		-4.38	-1.14	Large	**<0.001**

	Scar	12	45.8 (14.4)	58.3 (22.2)		-3.32	-0.89	Large	**0.007**
Peripheral vision	Atrophy+Scar	25	40.0 (14.4)	56.0 (19.5)	t Student	-5.63	-1.09	Large	**<0.001**
	Atrophy	13	42.3 (15.7)	57.7 (21.4)		-3.41	-0.89	Large	**0.005**
	Scar	12	37.5 (13.1)	54.2 (17.9)		-4.69	-1.26	Large	**<0.001**
